# Supplementary material for: The microbiome biomarkers of pregnant women’s vaginal area predict preterm prelabor rupture in Western China
Source: Front Cell Infect Microbiol. 2024 Oct 31;14:1471027. doi: 10.3389/fcimb.2024.1471027 (PMC11560878; doi:10.3389/fcimb.2024.1471027)
Supplement: Supplementary file 1 [file DataSheet1.zip › compare_1/Community/Structure_Barplot/genus/genus.taxon.Barplot.Legend.With.order.pdf]

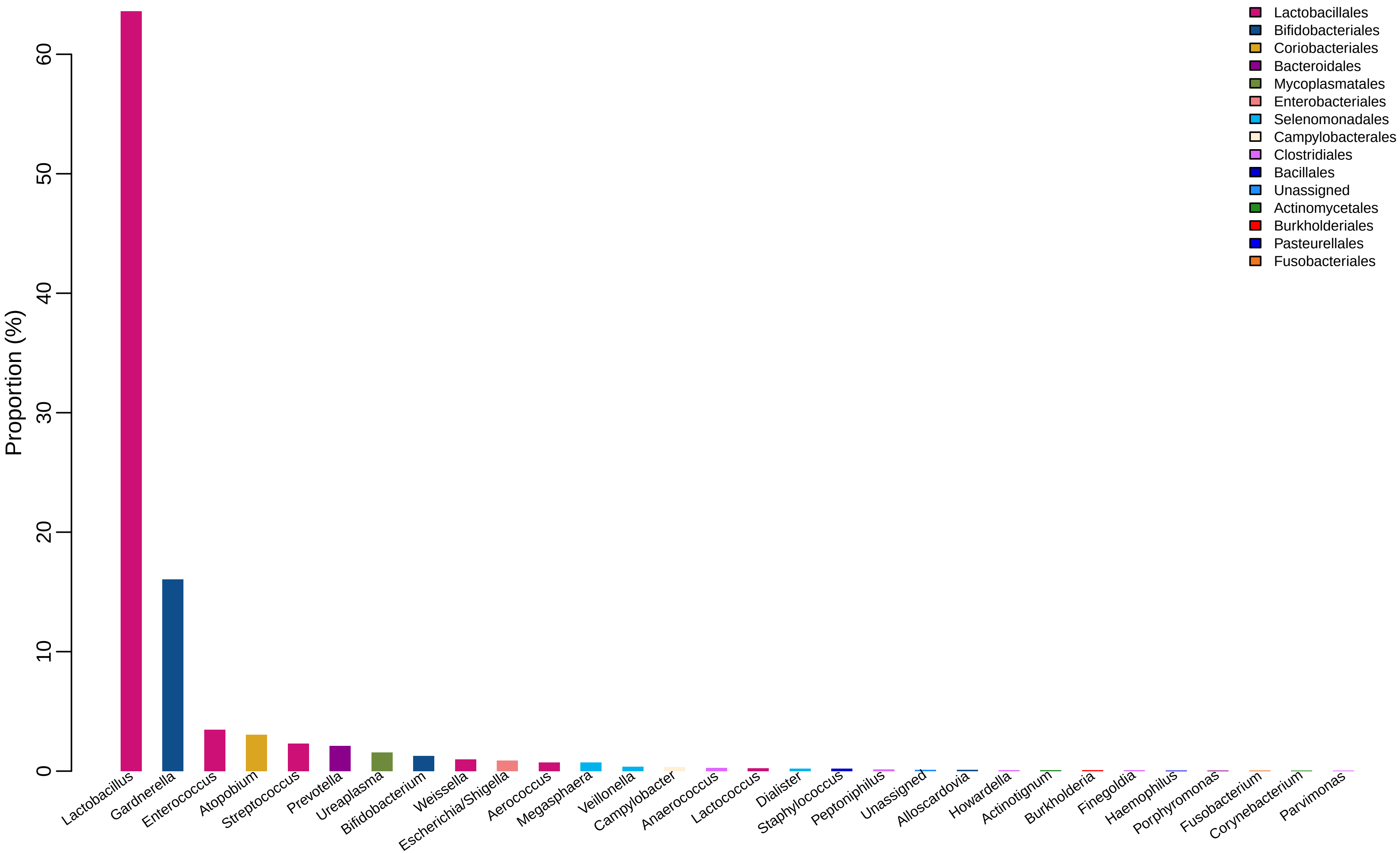

Proportion (%)
0
10
20
30
40
50
60
Lactobacillus
Gardnerella
Enterococcus
Atopobium
Streptococcus
Prevotella
Ureaplasma
Bifidobacterium
Weissella
Escherichia/Shigella
Aerococcus
Megasphaera
Veillonella
Campylobacter
Anaerococcus
Lactococcus
Dialister
Staphylococcus
Peptoniphilus
Unassigned
Alloscardovia
Howardella
Actinotignum
Burkholderia
Finegoldia
Haemophilus
Porphyromonas
Fusobacterium
Corynebacterium
Parvimonas
Lactobacillales
Bifidobacteriales
Coriobacteriales
Bacteroidales
Mycoplasmatales
Enterobacteriales
Selenomonadales
Campylobacterales
Clostridiales
Bacillales
Unassigned
Actinomycetales
Burkholderiales
Pasteurellales
Fusobacteriales
